# FGF-2 enriched nanofiber scaffold for advancing achilles tendon healing: a comparative experimental investigation

**DOI:** 10.3389/fsurg.2024.1424734

**Published:** 2024-10-17

**Authors:** Necmettin Turgut, Funda Cengiz Çallıoğlu, Aytül Bayraktar, Mehtap Savran, Halil Aşcı, Kanat Gülle, Meriç Ünal

**Affiliations:** ^1^ Department of Orthopedics and Traumatology, Faculty of Medicine, Adana Dr. Turgut Noyan Research and Training Centre, Başkent University, Adana, Türkiye; ^2^ Department of Textile Engineering, Engineering Faculty, Süleyman Demirel University, Isparta, Türkiye; ^3^ Department of Chemistry Engineering, Engineering Faculty, Süleyman Demirel University, Isparta, Türkiye; ^4^ Department of Pharmacology, Faculty of Medicine, Süleyman Demirel University, Isparta, Türkiye; ^5^ Department of Histology and Embryology, Faculty of Medicine, Süleyman Demirel University, Isparta, Türkiye; ^6^ Department of Orthopedics and Traumatology, Private Meddem Hospital, Isparta, Türkiye

**Keywords:** Achilles tendon, FGF-2, fibroblast growth factor, scaffold, tendon repair, tendon rupture

## Abstract

**Introduction:**

Achilles tendon rupture is a common and debilitating injury that significantly impacts mobility and quality of life. Effective treatment options that promote faster and more complete healing are needed. Fibroblast growth factor-2 (FGF-2) has shown potential in enhancing tendon repair. This study aims to investigate the efficacy of FGF-2 in promoting tendon healing in a rat model of Achilles tendon rupture, providing insights into its potential as a therapeutic option.

**Materials and methods:**

Forty-eight rat hind legs with complete Achilles tendon ruptures were divided into four equal groups: the Sham (S) group (tendon repair only), the Polymer (P) group (tendon repair with scaffold wrapping), the Produced FGF-2 (PF) group (scaffold coated with lab-produced FGF-2), and the Commercial FGF-2 (CF) group (scaffold coated with commercially sourced FGF-2). Histological analyses at two and four weeks post-surgery evaluated healing based on nuclear morphology, vascularity, fibril organization, inflammation, and adipogenesis.

**Results:**

At the end of the second week, no macroscopic healing was observed in one rat each from the S and P groups. By the end of the fourth week, macroscopic healing was observed in all groups. The S and P groups exhibited similarly severe fibril disorganization, pathological adipogenesis, and sustained inflammation, particularly at the fourth week. In contrast, the CF group demonstrated improved tendon healing with increased vascularity and extracellular matrix, lower inflammatory cell infiltration, and better fibril organization. Pathological adipogenesis was absent in the CF group, especially at the fourth week. The PF group showed comparable improvements at the second week but experienced a relapse by the 4th week, with increased inflammation and adipogenesis.

**Conclusion:**

FGF-2 coated scaffolds significantly enhanced tendon healing in a rat Achilles tendon rupture model by improving fibril organization, increasing vascularity, and reducing inflammation and pathological adipogenesis. These findings suggest that FGF-2 could be a promising therapeutic option for accelerating tendon repair. Future perspectives on tendon repair will focus on enhancing FGF-2 delivery using innovative scaffolds, paving the way for more effective therapies and improved patient outcomes.

## 1 Introduction

The Achilles tendon, despite being one of the most robust tendons in the human body, is paradoxically the most commonly ruptured tendon in the lower extremities (1). Achilles tendon ruptures occur at an estimated rate of 10 per 100,000 people annually, making it a significant concern in orthopedic and sports medicine (2). Among ankle injuries, Achilles tendon rupture presents a particular challenge, and there remains ongoing debate about whether conservative or operative treatment should be the preferred approach. Currently, no consensus exists on the superiority of conservative vs. operative treatment for managing this injury (3, 4). However, higher rates of rerupture and reduced ankle range of motion associated with conservative treatment make surgery the predominantly chosen primary option (5). Surgical treatment is especially preferred in athletes, young individuals or high-demand workers (6, 7). Studies on this issue continue to be conducted with the goal of helping patients return to their previous functional status and professional activities more quickly.

Patients may experience issues due to the insufficient quality of the repaired tendon or prolonged recovery period, even with surgical treatment which can lead to dissatisfaction. Therefore, all efforts have been focused on achieving tendon characteristics similar to the original strength and power of the native tendons in a shorter time frame. However, with current treatment methods, whether conservative or surgical, restoring the tendon to the same biomechanical strength or identical characteristics as the unaffected tendon remains unattainable. Efforts are underway to improve the healing process through the use of injectable regenerative biomaterials or therapeutics that can be implanted during open surgery.

Research into tissue engineering has gained momentum in across various medical fields, including the treatment of tendon injuries. Bioengineered scaffolds and growth factors have also been investigated for their role in tendon healing in recent years (8). Scaffolds are one of the safe and efficient ways to maintain growth factors at high and sufficient concentrations during tendon healing. They provide extra support as carriers for other healing factors and create a biomimetic environment for repair. Several growth factors have been proposed to promote ligament and tendon healing: vascular endothelial growth factor (VEGF), epidermal growth factor (EGF), transforming growth factor-β (TGF-β), and basic fibroblast growth factor (bFGF or FGF-2) (9). FGF-2 is particularly valuable factor as it promotes mitosis, induces angiogenesis, chondrogenesis, and also promotes cell differentiation and proliferation of mesenchymal stem cells (10–12). FGF-2 has been shown to be an important contributor for tendon, ligament healing, cartilage repair, and bone repair (13). Due to these characteristics, growth factor-based treatments are being explored for their effects on damaged tendons and ligament structures.

Despite the promising potential of growth factors like FGF-2, research on the effectiveness of combining FGF-2 with scaffolds for Achilles tendon repair is limited. This study aims to address this gap by investigating the usefulness and efficacy of FGF-2, produced using a newly designed gene profile by our research group, and commercially obtained FGF-2 when added to scaffolds in a rat Achilles tendon rupture model with histological analyses. Our hypothesis is that scaffolds loaded with FGF-2 will improve healing outcomes in Achilles tendon ruptures.

## 2 Materials and methods

### 2.1 Production of FGF-2

FGF-2 was synthesized for this project using the methodology outlined in Kürkçü's master's thesis (14). The study demonstrates the successful expression of human FGF-2 protein in Escherichia coli using the BL21 (DE3) expression system to produce pET32a-*E. coli* FGF-2. Enhanced productivity and optimization were achieved by co-expressing *E. coli* with thioredoxin. This study adapts the methodology pioneered by Soleyman et al. (15). An S-tag was used as the fusion protein instead of a histidin-tag (his-tag). Polymerase chain reaction (PCR) primer sequences were designed to isolate and amplify the FGF-2 gene from the human genome. These sequences were exploded using the online software “https://blast.nlm.nih.gob/Blast.cgi”. In order to obtain site specificity, primers of suitable length and specification were designed. The FGF-2 synthetic gene was PCR amplified [ATG + TRX + S.TAG + DDDDK + (gly 4 Se) 2 + FGF-2 + UAA]. S-Tag sequence is a new S-tag detection and high precision purification makes it easily testable. Its small size, low cost, and specificity offer additional advantages to S-protein over other enzyme-based determinations. Additionally, S-protein-based reagents can be detected in sodium dodecyl sulfate-polyacrylamide gel electrophoresis (SDS-PAGE) blots for S-tag fusions using colorimetric or chemiluminescence detection of the target protein up to nanogram quantities.

### 2.2 Production of nanofiber scaffold

#### 2.2.1 Materials

In this study, a Polyurethane/dimethylformamide (PU/DMF) and polycaprolactone/dimethylformamide:chloroform (2:8) (PCL/DMF:CHL) based double layered nanofiber scaffold was produced by electrospinning. PCL (Mn 80.000) and PU (Pellethane 2103-80AE) were used as the polymers, while DMF and chloroform served as the solvents. PCL polymer and solvents were purchased from Sigma-Aldrich Corporation (St. Louis, MO, USA), and the PU polymer was supplied by Velox- Lubrizol Advanced Materials. PCL and PU polymer solutions were prepared at concentrations of 7 wt% and 12 wt%, respectively. All solutions were prepared under consistent conditions including stirring time, stirring speed, and temperature.

#### 2.2.2 Methods

The methods were planned according to the study by Cengiz Çallıoğlu et al. (16). First, PCL/DMF:CHL and PU/DMF polymer solutions were prepared. Then, polymer solution properties were determined, such as: viscosity (Lamy Rheology, B-One Touch Screen) under a shear rate of 5 s^−1^, conductivity (SelectaCD2005), and surface tension according to the Wilhelmy Plate method (Biolin Scintific Sigma 702). Each measurement was taken three times.

Afterwards, nanofibers were prepared using conventional laboratory-scale needle electrospinning. Table 1 shows electrospinning process parameters during the spinning process for PU and PCL. All nanofibers were collected on aluminum foil. Both PCL and TPU polymer solutions were spun half an hour.

**Table 1:** Translation of the process parameters applied in electrospinning process.

Polymer	Feed rate (mL/h)	Voltage (kV)	Needle diameter (mm)	Distance (cm)	Temperature (°C)	Humidity (%)
PU	0.9	19.9	0.8	20	24,9	35
PCL	0.5	18	0.8	18	23	45

PU, polyurethane; PCL, polycaprolactone.

The morphology of electrospun nanofibers was examined using scanning electron microscopy (SEM) on a FEI Quanta 250 FEG model instrument. Images were captured at 1.000× and 10.000× magnifications. Electrospun web quality was assessed at 1.000×, 5.000×, while fiber diameters were measured at 10.000×. Fiber diameters were measured using ImageJ software, based on measurements of 50 fibers taken from different parts of the electrospun web.

### 2.3 Nanofiber scaffold characterization

First, the solution properties of PU/DMF and PCL/DMF:CHL were determined. The results for conductivity, viscosity, and surface tension are shown in Table 2. From the results; it was found that conductivity, viscosity, and surface tension values of the PU/DMF solution were higher than those of the PCL/DMF:CHL solution.

**Table 2:** Solution properties of PU/DMF and PCL/DMF: chloroform.

Sample	Conductivity (μS/cm)	Viscosity (Pa.s)	Surface tension (mN/m)
12 wt % PU	1.10	1.341	34.64
7 wt % PCL	0.24	0.436	28.86

**Figure 1:**
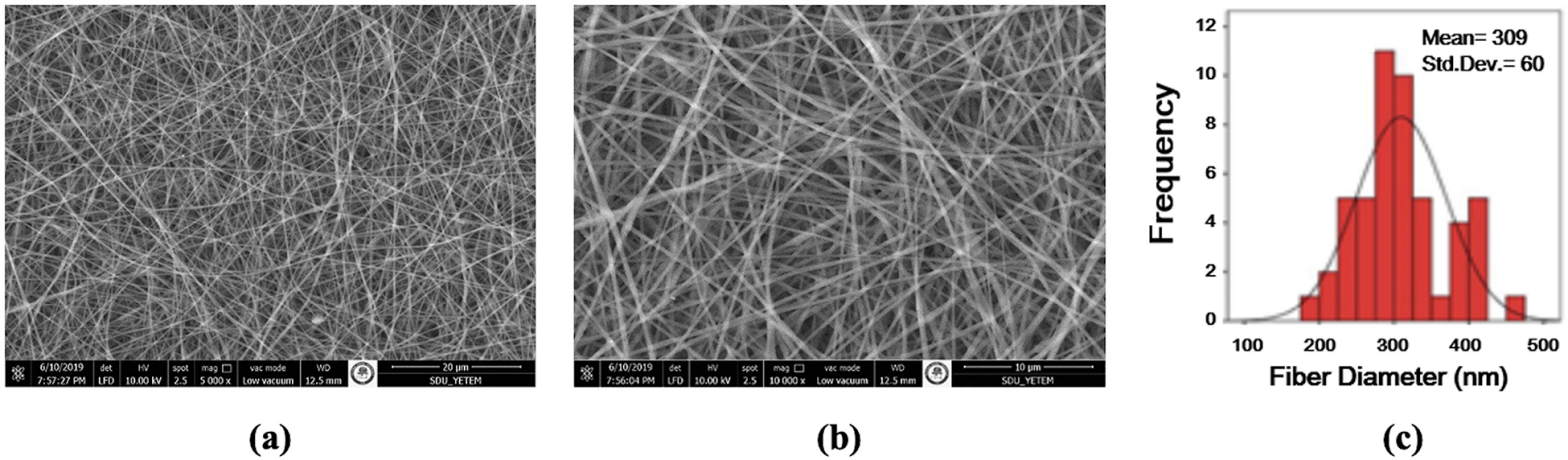
SEM images of 12 wt % PU/DMF nanofibers and fiber diameter histogram **(a)** 5.000× **(b)** 10.000×), **(c)** diameter histogram.

**Figure 2:**
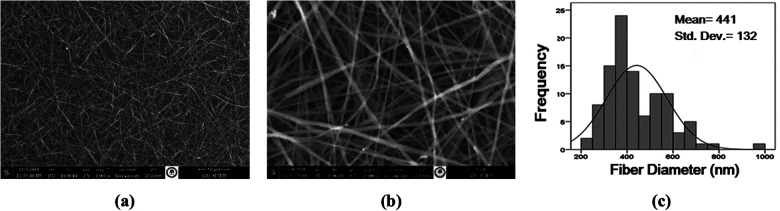
SEM images of 7 wt % PCL/DMF: chloroform nanofibers and fiber diameter histogram **(a)** 1.000× **(b)** 10.000×), **(c)** diameter histogram.

Figures 1, 2 show the SEM images of nanofiber scaffolds and the fiber diameter histograms for both PU and PCL, respectively. The average fiber diameters were calculated as 309 nm (PU) and 441 nm (PCL) using ImageJ analysis software. SEM images revealed that a bead-free nanofiber scaffold, consisting of very thin fibers with a high degree of porosity, was produced. Additionally, the fiber diameter histograms showed a unimodal curve, indicating that uniform nanofibers were obtained in this study.

**Figure 3:**
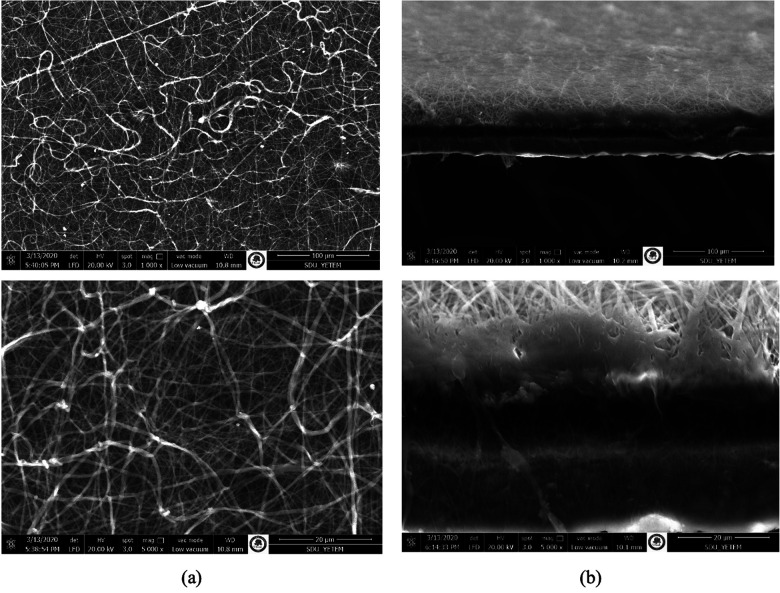
SEM images of PU/PCL nanofibers **(a)** fiber morphology (1.000×, 5.000×), **(b)** cross-section of bilayer of nanofibers (1.000×, 5.000×).

On the other hand, SEM images of double layer PU and PCL nanofibers morphology and cross-section are presented in Figure 3. The double-layer product was cut into equal sizes, sterilized, and the inner surface was coated with FGF-2. Due to stability concerns, FGF-2 was applied onto the nanofiber scaffold just before the experiment.

### 2.4 Experiment

After obtaining approval from the local Animal Experiments Local Ethics Committee, forty-eight adult male rats (weighing 400–425 g and older than 10 weeks) were randomly divided into four groups, with twelve rats in each group. The right hind legs were used in the experiments. Each group was treated in a different way for the Achilles tendon rupture model:

1. Sham group (S): Only primary tendon repair was performed after creation of Achilles tendon rupture model.
2. Polymer group (P): A polymer was added to primary tendon repair after creation of Achilles tendon rupture model.
3. Produced FGF-2 group (PF): Produced FGF-2 coated scaffold was wrapped around the primary tendon repair area after creation of Achilles tendon rupture model.
4. Commercial FGF-2 group (CF): Commercial FGF-2 coated scaffold was wrapped around the primary tendon repair area after creation of Achilles tendon rupture model.

#### 2.4.1 Surgical procedure

The rats were anesthetised with 10% ketamine HCl (50 mg/kg IM, (Alfamine®) and 2% xylazine HCl (5 mg/kg IM, (Rompun®). For prophylactic purposes, ceftriaxone (50 mg/kg, Rocephin®) was administered via intramuscular injection half an hour before and 12 h after the surgical procedure. Aseptic surgical conditions were ensured using povidone-iodine (Baticon®) after shaving the surgical area. A posterior approach to the right hind legs of the rats was used to prepare the model. A two-centimeter longitudinal incision was made, exposing the tendon after passing through soft tissue. A complete tenotomy of the Achilles tendon was performed using a No.15 scalpel blade, with a horizontal cut made one centimeter proximal to the calcaneal insertion. The plantaris tendon was also cut to prevent any pontential internal splinting effect. The transected ends of the tendon were repaired in all rats using 3/0 Prolene (Propylene®, Doğsan, Turkey) suture with a Kessler stitch.

**Figure 4:**
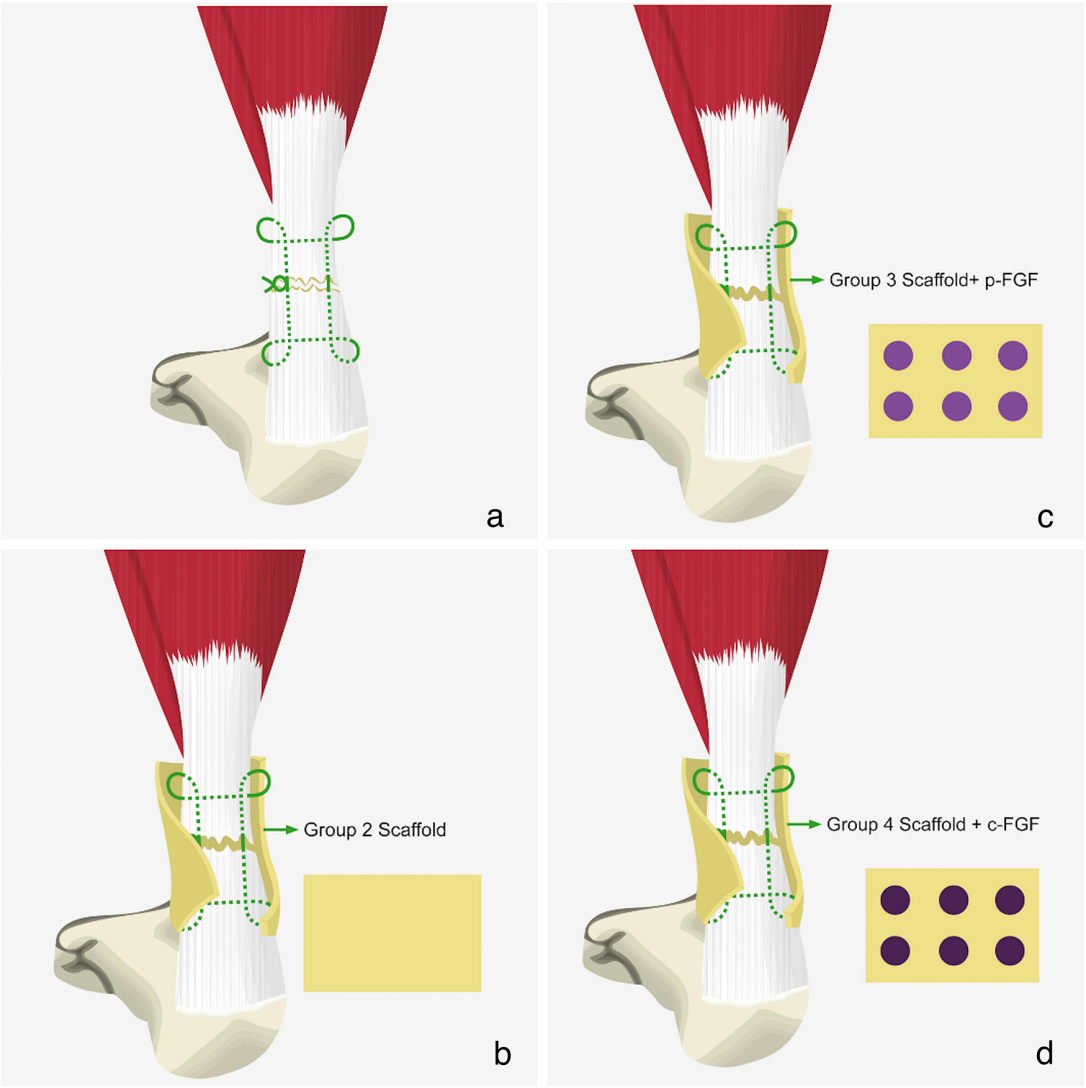
**(a)** Group 1 received only achilles tendon repair. **(b)** Group 2 received Achilles tendon repair plus scaffold. **(c)** Group 3 received Achilles tendon repair plus scaffold, with the addition of p-FGF. **(d)** Group 4 received Achilles tendon repair plus scaffold, with the addition of c-FGF.

**Figure 5:**
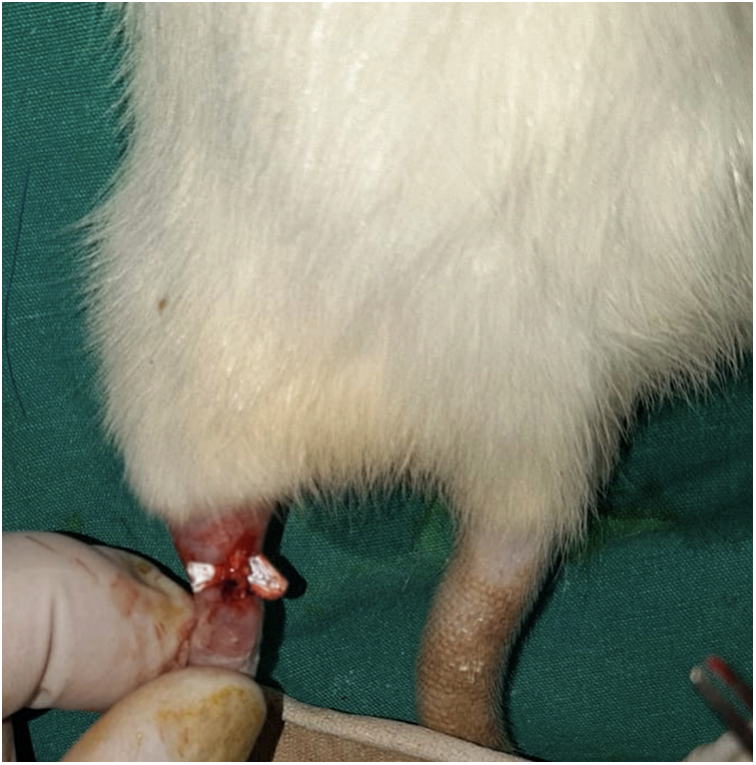
End of the procedure before skin closure.

The rats were grouped according to the treatment methods applied to their repaired Achilles tendons (Figure 4). No further treatment was applied to the S group. The scaffolds were wrapped around the repaired Achilles tendons in the other three groups (Figure 5). In Group P, a scaffold without FGF-2 was wrapped around the tendon. In Group PF, a scaffold containing FGF-2 produced by our team was wrapped around the tendon. In Group CF, a scaffold containing commercially obtained FGF-2 was wrapped around the tendon. The skin was closed with silk sutures in all rats.

Support for heat and fluid loss was provided to the animals after their wounds were dressed. Following the experimental surgeries, each rat was placed in a separate cage and monitored under pre-determined standard conditions. The rats were free to bear weight and no cast was applied. Additional dressings or analgesic treatments were not routinely administered. The need for pain relief was assessed by observing the routine movements and nutritional characteristics of the animals. The rats were allowed to feed freely during their 12-h light/dark cycle.

Six rats from each group were sacrified under xylazine HCl and ketamine HCl anesthesia at the end of the second week post-surgery, and the tendon samples were harvested using the same approach to the hind legs. The remaining six rats in each group were monitored under the same conditions and were sacrificed at the end of the fourth week. Tissue samples were preserved in formaldehyde solution for the histological evaluation of tendon healing using hematoxylin-eosin staining.

#### 2.4.2 Histological analysis

Animal tendons were fixed in a 10% neutral formaldehyde solution for at least 48 h. After fixation, the tissue samples were washed overnight in running water. The washed samples were then dehydrated through a series of alcohol solutions, clarified with xylene, and embedded in paraffin. Sections of 4–5 μm thickness were obtained from the paraffin blocks using a microtome (RM2125RTS, Leica, Germany). Sections from all groups were stained with hematoxylin-eosin for histopathological evaluation. Different areas at 40× magnification were examined for each section. Tissues were evaluated for nuclear morphology, vascularity, fibril disorganization, inflammatory cell infiltration, and pathological adipogenesis. For each animal, cell counting was performed on 10 different areas of tissue sections. The number of cells was determined. Histopathological scoring was based on the degree of findings. The results are presented in Table 3. Photographs of the sections were taken using a digital binocular light microscope (ECLIPSE Ni-U, Nikon, Tokyo, Japan).

**Table 3:** Evaluation of histopathological parameters of animal experiment.

Parameter/group		S group	P group	PF group	CF group	*p*
	Mean ± SD					
Cell nucleus morphology (oval/elongated)	2nd week	17.67 ± 2.07	17.00 ± 1.67	17.5 ± 2.26	18 ± 2.61	0.882
	4th week	12.67 ± 1.63	12.83 ± 1.33	17.67 ± 1.21	13 ± 2.10	**<0** **.** **001** *
	*p*	**0.002** *	**0.007** *	0.842	**0.017** *	
Vascularity	2nd week	17.17 ± 1.17	16.83 ± 0.98	34.0 ± 2.61	50.5 ± 2.74	**<0** **.** **001** *
	4th week	16.33 ± 1.51	17.17 ± 1.32	34.33 ± 2.66	17.0 ± 1.27	**0** **.** **003** *
	*p*	0.343	0.671	0.914	0.317	
Fibril disorganization	2nd week	30.67 ± 1.75	19.83 ± 2.71	10.33 ± 1.63	9.67 ± 1.63	**<0** **.** **001** *
	4th week	20.17 ± 1.47	20.17 ± 2.23	20.17 ± 2.93	9.83 ± 2.04	**0** **.** **004** *
	*p*	**0.026** *	0.833	**0.024** *	0.783	
Inflammatory cell infiltration	2nd week	19.33 ± 2.5	19.67 ± 2.58	10.17 ± 0.75	10.17 ± 2.14	**0** **.** **001** *
	4th week	20.0 ± 0.63	20.17 ± 1.47	20.17 ± 1.94	10.0 ± 1.10	**0** **.** **004** *
	*p*	0.498	0.915	**0.028** *	0.666	
Pathological adipogenesis	2nd week	20.17 ± 3.19	10.0 ± 1.27	0.33 ± 0.52	0.17 ± 0.4	**<0** **.** **001** *
	4th week	29.83 ± 1.47	19.83 ± 1.17	19.83 ± 0.98	0	**<0** **.** **001** *
	*p*	**0.027** *	**0.027** *	**0.024** *	**0.027** *	

* Bold values indicate statistically significant differences (*p* < 0.05). *Post hoc* LSD tests indicated the following significant differences:
• Cell nucleus morphology:
○ 2nd week: No significance. ○ 4th week: Significant between S group and PF group; P group and PF group; CF group and PF group. • Vascularity:
○ 2nd week: Significant between S group and CF group; P group and CF group. ○ 4th week: Significant between S group and PF group; P group and PF group; CF group and PF group. • Fibril disorganization:
○ 2nd week: Significant between S group and CF group; S group and PF group. ○ 4th week: Significant between S group and CF group; CF group and PF group; P group and CF group. • Inflammatory cell infiltration:
○ 2nd week: Significant between PF group and P group; PF group and S group; CF group and P group; CF group and S group. ○ 4th week: Significant between CF group and S group; CF group and PF group; CF group and P group. • Pathological adipogenesis:
○ 2nd week: Significant between S group and CF group; S group and PF group. ○ 4th week: Significant between S group and CF group.

## 3 Results

### 3.1 Macroscopic assessment

At the end of the fourth week, it was observed macroscopically that the healing in the groups containing FGF-2 was better in animals. Complete tendon healing was observed in all rats in the CF and PF groups at both time points. However, one rat in each of the S and P groups did not show macroscopic healing at the second week, although healing was complete by the fourth week.

### 3.2 Histological assessment

**Figure 6:**
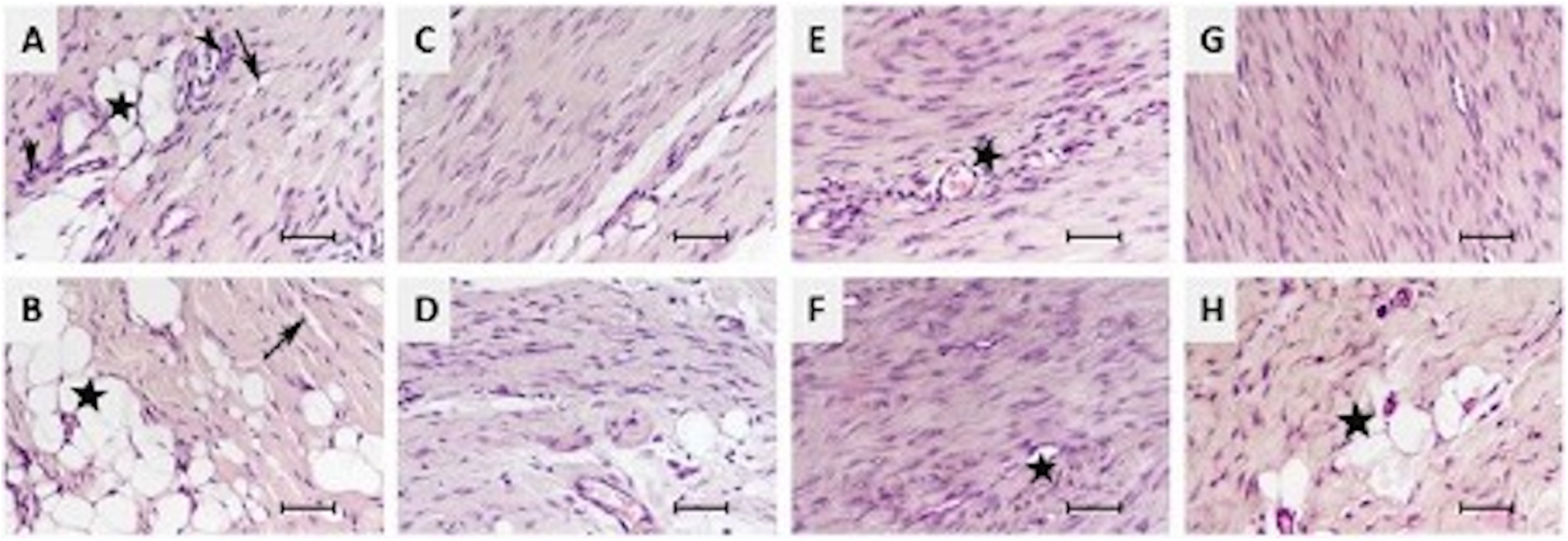
Histopathological results of the animal experiment. **(A,B)** Sham group at 2nd and 4th weeks; widespread pathological adipogenesis (black stars), disruption of fibril organization (black arrows), edema, and inflammatory cell infiltrations (black arrowheads). **(C,D)** Polymer group at 2nd and 4th weeks showing similar findings to the sham group. **(E,F)** CF group at 2nd and 4th weeks demonstrating increased vascularity and inflammatory cell infiltrations (black stars). **(G,H)** PF group at 2nd weeks showing increased vascularity and inflammatory cell infiltrations, and at 4th weeks, these findings were accompanied by pathological adipogenesis (black star). HE, ×40, scale bar = 50 µm.

In the second-week sections of the S group, numerous oval-nucleated cells, widespread pathological adipogenesis, fibril disorganization, edema, and inflammatory cell infiltrations were observed (Figure 6A). Fibril disorganization was significantly higher in the S group compared to the PF and CF groups at the second week (*p* < 0.001). By the fourth week, it was observed that the pathological adipogenesis areas and elongated-nucleated cells increased, along with the persistence of other findings (Figure 6B).

Similar findings were detected in the P group's second and fourth-week sections (Figures 6C,D).

In the CF group's second-week sections, numerous oval-nucleated cells and a significant increase in vascularity were observed. Pathological adipogenesis and edema were absent, although inflammatory cell infiltrations were present (Figure 6E). By the fourth week, in addition to findings similar to the second week, an increase in extracellular matrix and elongated-nucleated cells was noted (Figure 6F). At the fourth week, the CF group showed significantly better fibril organization, less inflamatuar cell infiltration, and less pathologic adipogenesis compared to the other groups (*p* = 0.004, *p* = 0.004, and *p* < 0.001, respectively).

In the PF group's second-week sections, similar findings to those in the CF group's second-week sections were observed (Figure 6G). However, by the fourth week, an increase in inflammatory cell infiltrations and the presence of pathological adipogenesis were detected (Figure 6H). Cell nucleus morphology (oval/elongated) and vascularity were significantly higher in the PF group compared to the other groups in the fourtth-week sections (*p* = 0.003).

All findings are summarized in Table 3.

## 4 Discussion

The most important finding of the present study is that the application of an FGF-2 coated scaffold Achilles tendon rupture can enhance the quality of the repair tissue and improve the structural organization. We found that this treatment method was associated with increased vascularity, extracellular matrix production, and improved cell proliferation. These findings align with previous research (17, 18). Chan et al., in a rat patellar tendon model, found that increased cell proliferation and expression level of type III collagen were obtained by FGF-2 injections at first week postinjury, although they could not provide evidence of biomechanical positive effects on ultimate stress (19). Other studies using canine and rabbit models have also shown that FGF-2 levels were particulary elevated in the early phase of tendon healing (20, 21). Hamada et al. developed a monofilament nylon suture that released FGF-2 during flexor tendon repair, which resulted in increased biomechanical strength and a thicker epitenon by the end of the third week (22). The term “epitenon” refers to the connective tissue sheath surrounding the tendon, which plays a crucial role in tendon healing by providing structural support and facilitating the repair process. The study also showed that FGF-2 levels were elevated during the first week when administered exogenously. After this initial increase, FGF-2 levels decreased and stabilized at a consistent level throughout the later stages of healing. This result may explain why inflammatory infiltration and pathologic adipogenesis were observed in the PF group at the fourth week, even though none of these findings were absent at the second week. On the other hand, some researchers have reached opposite conclusions. Kraus et al. found increased expression of type I collagen in an *in vivo* rat model based on stem cells and stable lentiviral FGF-2 expression. However, none of the expected results were observed in the later stage biomechanical tests (23, 24). A possible explanation of for the inefficacy of FGF-2 application could be due to the created tendon-defect model instead of a transection model. Kraus et al. further investigated whether FGF-2 had any histological or biomechanical benefits at the end of the 12th week, but they did observe hardly any effects (24).

Significant positive results were observed in both the PF and the CF groups compared to the S and P groups in our experimental study. Complete tendon healing was noted in all rats treated with FGF-2. However, macroscopic healing was not observed in one rat from the S and P groups at 2 weeks. By 4 weeks, tendon healing was evident in all rats. Histological parameters were the primary outcome measures in our study. The unfavorable histopathological findings observed in the S and P groups at 2 weeks were also observed to persist at 4 weeks. While the produced FGF-2 group showed tendon healing quality similar to the CF group, an increase in inflammatory cell infiltrations and the presence of pathological adipogenesis were detected at 4 weeks.

The positive results obtained with PF in the early stages of Achilles tendon healing, particularly in the second week, are promising for future tendon characteristics. This is clinically significant, because most reruptures of Achilles tendons occur early within the first weeks following treatment (25). We assume that the treatment will be beneficial for early return to activity in athletes, allowing for the start of early rehabilitation in athletes and the prevention of rerupture, particularly in patients with tendon defects, through the use of FGF-2 loaded scaffolds. In a recent study, a polycaprolactone scaffold loaded with FGF-2 was shown to exhibit greater stiffness and higher peak load compared to the native ACL in an athymic rat model (26). Positive effects of FGF-2 were also observed in a canine model, where it contributed to improved ACL maturation (27).

FGF-2 influences tendon healing through several key mechanisms. It stimulates fibroblast proliferation and activation, which enhances collagen synthesis and improves the quality of the extracellular matrix (18). This effect is mediated by FGF-2 binding to its receptor, leading to the activation of intracellular signaling pathways such as ERK1/2 and PI3K/Akt, which promote cell division and matrix production (28). Additionally, FGF-2 promotes angiogenesis, supporting tissue repair by increasing blood supply (29). It also helps regulate the inflammatory response, creating a more favorable environment for healing (30). These mechanisms collectively contribute to the therapeutic potential of FGF-2 in tendon repair. FGF-2, a mitogenic polypeptide, was initially identified in the pituitary gland of the bovine brain (31). Due to its limited availability, isolation challenges, and high demand for large quantities, FGF-2 needs to be highly expressed and purified for various applications such as tissue regeneration, wound healing, and stem cell research (32). Various hosts, including *E. coli*, *Bacillus subtilis*, *Pichia pastoris*, soybean seeds, silkworm (*Bombyx mori* L.), and rice seeds, have been used for the production of recombinant FGF-2 (33, 34). In this study, a newly designed gene profile, not previously mentioned in the literature, was utilized for the expression of recombinant FGF-2 protein at high purity and quantity, leveraging bioinformatics in the *E. coli* expression system. This system offers several advantages, including cost-effectiveness, time efficiency, ease of culture, rapid growth, and simple recovery of recombinant protein when expressing a heterologous recombinant protein (14). To improve the solubility of FGF-2, a Trx tag was employed in conjunction with the pET-32a(+) expression vector system. Additionally, an S. Tag was incorporated between the Trx and FGF-2 coding sequences for affinity chromatography detection and rapid purification. The S. Tag system relies on a strong interaction between the 15-amino acid S. Tag and the 103-amino acid S-protein, both derived from RNase A, which together reconstitute ribonucleolytic activity.

In a study by Soleyman et al., recombinant FGF-2 productivity was evaluated by producing it in different growth media with recombinant *E. coli* BL21 cells at various incubation temperatures to determine the optimal conditions (15). Similarly, in our study, the optimal incubation temperature was found to be 37°C. TB and LB media showed high productivity due to the presence of yeast extract, tryptone, peptone, and phosphates. Additionally, the use of glycerol as a carbon source and a phosphate buffer helped mitigate adverse pH changes (15). This study found that TB medium demonstrated better production potential than LB medium. In contrast to this study, Gasparian et al. used BglII and HindIII restriction enzymes (35). The *E. coli* strain BL21 (DE3) was transformed with the DNA pET-32a/FGF-2 plasmid, and the bacteria were grown in LB and TB media, harvested, and analyzed using SDS-PAGE. Only Trx was used as a fusion protein, producing a single band of approximately 17 kDa on polyacrylamide gel. In this study, Trx and S. Tag fusion proteins were used, along with DDDDK and (Gly4Se2). Upon induction with PCR, the FGF-2 protein produced a band image of 24 kDa in terms of production potential. As a result, a recombinant FGF-2 protein with a new gene profile was successfully developed, offering commercial production capability, patentability, high purity, and yield. The production method of this protein is expected to pave the way for the development of different recombinant protein production techniques, further enhancing the significance of the study, as the produced protein can be applied in the health, biomedical, and cosmetic fields.

Tendon tissue engineering offers a promising approach for tendon ruptures. An ideal scaffold creates should mimic the native extracellular matrix of the applied tendon or ligament and possess the mechanical chararacteristics necessary to provide structural support for tendon healing (17). Like other growth factors, FGF-2 has a short plasma half-life, requiring repeated local applications in fact. Therefore, it is crucial to sustain the release of growth factors over a prolonged period within a mechanically strong structure. To achieve this, nanofiber scaffolds using electrospinning technology have been developed (36). The P group was investigated whether it had positive effects on tendon healing independently, but no significant differences were found than those of S group in the present study. This highlights the importance of incorporating therapeutic factors into the scaffold to enhance tendogenesis. Although we chose to investigate the FGF-2 growth factor independently, it is well known that growth factors can be used in combination within scaffolds to produce synergistic effects and improve healing. A combination of IGF-1, PDGF-BB, FGF-2 was found to be associated with increased cell proliferation in a study (37).

The use of nanometer-sized fibrous scaffolds in our study contrasts with the micrometer-sized PCL scaffolds reported in previous research (38). This comparison highlights significant differences in scaffold properties and their potential impacts on tissue regeneration. Nanometer-sized scaffolds, as used in our study, offer several advantages including closer mimicry of the extracellular matrix at a cellular scale, which enhances cell adhesion, proliferation, and differentiation. The increased surface area and porosity of nanofibers also facilitate improved growth factor delivery and cellular interaction, which are critical for effective tendon repair and regeneration. Conversely, micrometer-sized scaffolds, as reported in the referenced study, provide superior mechanical support and stability. They are generally easier to fabricate and handle, offering more robust structural integrity in load-bearing applications.

This study demonstrates that the use of FGF-2, particularly the commercial variant, significantly enhances tendon healing by promoting better fibril organization, increased vascularity, and reduced inflammatory responses and pathological adipogenesis. While both the CF and PF groups showed promising improvements at the second week, the CF group exhibited more consistent and sustained healing benefits by the fourth week. In contrast, the S and P groups showed less favorable outcomes, with persistent inflammation and tissue disorganization. These findings highlight the therapeutic potential of FGF-2 in enhancing tendon repair and suggest that further exploration of growth factor-based interventions could lead to more effective treatments for Achilles tendon rupture.

Future research needs to address several critical areas to fully harness FGF-2's potential in tendon tissue engineering. These include clarifying FGF-2's synergistic and antagonistic interactions with other growth factors and therapeutic components, as well as determining the optimal timing and duration for its delivery. Additionally, optimizing its dose to maximize healing while minimizing risks such as fibrosis and adhesion is crucial for effective clinical application. Another important focus is the use of biomaterial scaffolds for sustained FGF-2 release. These scaffolds could enhance the long-term effects of FGF-2 by promoting neovascularization and better tissue integration. Furthermore, combining FGF-2 with stem cell therapies could offer even greater promise for tendon healing by allowing stem cells to mediate repair and continuously produce regenerative stimuli over time. Advancements in scaffold design, gene therapy, and recombinant growth factors will be crucial in refining these strategies and overcoming the challenges of tendon repair in the future. Future research should also focus on transitioning from animal studies to clinical trials to assess the potential of FGF-2 in humans, with an emphasis on long-term outcomes and recovery quality. Exploring the combination of FGF-2 with stem cell therapies or advanced scaffold technologies may further enhance healing (39, 40). This direction could pave the way for developing commercially viable therapies for tendon injuries.

This study is not out of limitations. First, one of the shortcomings can be shown that the lack of molecular and biomechanical evaluation, and as an animal experiment, it inherently does not fully reflect the human physiology. The sample size of rats was small and only hematoxylin-eosin staining was used for histopathologic evaluation. In this study, Achilles tendon healing in rats was examined at the end of two and four-week periods in agreement with the literature, longer observation periods after surgery would be beneficial for assessing the long-term effects of growth factors. Future studies should address these aspects for a more comprehensive understanding.

## 5 Conclusions

In conclusion, this study demonstrates the promising role of FGF-2 coated scaffolds in enhancing tendon healing by improving fibril organization, increasing vascularization, and reducing inflammation. Future research should focus on addressing the limitations of this study, including long-term evaluations, and the transition from animal models to clinical trials, to fully realize the potential of FGF-2 in clinical applications for tendon injuries.

## Data Availability

The original contributions presented in the study are included in the article/Supplementary Material, further inquiries can be directed to the corresponding author.
